# The Game Within the Game: The Potential Influence of Demand Characteristics and Participant Beliefs in Violent Video Game Studies

**DOI:** 10.1177/19485506241273193

**Published:** 2024-08-27

**Authors:** Yashvin Seetahul, Tobias Greitemeyer

**Affiliations:** 1 University of Innsbruck, Austria

**Keywords:** demand characteristics, demand effects, suspicion, bias, motivated responding, violent video games, aggression

## Abstract

In two experiments, we examined the potential impact of demand characteristics in violent video game (VVG) research. Study 1 (*N* = 788) measured behavioral aggression, while Study 2 (*N* = 1,182) measured trait aggression. Participants were informed either that researchers wanted to confirm that VVGs increase aggression (“Positive Hypothesis”) or that VVGs have no effect (“Null Hypothesis”). Study 2 included a third condition where participants were given no information. In both studies, the interaction between VVG exposure and experimental conditions was significant. Whereas VVG exposure was significantly positively associated with aggression in the “Null Hypothesis” condition, it was not in the “Positive Hypothesis” condition. These effects were driven by habitual players responding differently based on the presented hypothesis, appearing less aggressive in the “Positive Hypothesis” condition than in the other two conditions. These findings highlight the importance of addressing demand characteristics in VVG studies.

Since the 1970s, psychologists have extensively studied the relationship between violent video games (VVGs) and aggressive behavior (Kocurek, 2012). Using diverse research methods, including observational, longitudinal, and experimental designs, the impact of both short-term and prolonged VVG exposure have been examined and a substantial body of research has been produced, as evidenced, for example, by a meta-analysis by Anderson et al. (2010) that included 381 studies with more than 130,000 participants. The cumulative evidence suggests that VVGs lead to increased aggression, but effect sizes are typically no stronger than *r* = .20 (Anderson et al., 2010; Greitemeyer & Mügge, 2014; Mathur & VanderWeele, 2019; see also Greitemeyer, 2022). However, a clear consensus remains elusive (de Vrieze, 2018; Mehta & Lakens, 2023), and some researchers argue that VVG research is hampered by methodological flaws and publication bias, leading to an overestimation of the actual effect (e.g., Hilgard et al., 2017). Here, we make the case that one concern that remains largely overlooked is the impact of demand characteristics that may instead lead to an underestimation of the relationship between VVGs and aggression in some studies. In fact, with two experiments, we demonstrate that the interaction between participants’ beliefs about the study and their level of exposure to VVGs can negatively influence the outcome.^1^

## Insufficient Attention to Demand Characteristics

Demand characteristics, as defined by Orne (1962), are the totality of cues that may indicate the researcher’s goals to participants and, in turn, influence participants’ responses. These influences, referred to as “demand effects”, can compromise the validity of studies by creating empirical artifacts. Demand characteristics may be present within or surrounding a study, as they represent *any* element that provides information that can influence participants’ beliefs about the goal of the study. They refer to cues present in how studies are presented, how individual tasks are introduced, how the instructions are given, how a study is advertised, and any other place where participants may obtain information about the study. Despite their historical importance in psychology (e.g., Campbell, 1950), focus on demand effects has faded (see Sharpe & Whelton, 2016). Researchers often use conventional methods without considering their susceptibility to demand effects (Gergen, 1973; Klein et al., 2012). However, recent publications underscore a renewed interest in this topic (e.g., Coles et al., 2023; Corneille & Lush, 2023; Olson & Raz, 2021; Rubin, 2016). A recent meta-analysis (Coles & Frank, 2023) indicated high variability of demand effects across studies, highlighting the need to examine this mechanism in different research domains (see also Atwood et al., 2022; Boot et al., 2013; Dienes et al., 2022; Klein et al., 2012; Lush, 2020; Olson & Raz, 2021).

In VVG research, the issue of demand characteristics has often been relegated to brief, unverified assumptions used to justify methodological choices. For example, some researchers have measured covariates at the end of studies to reduce suspicion and avoid influencing the main outcome (e.g., Anderson et al., 2004, Exp. 3), or employed unrelated measures to minimize participant awareness (e.g., Tear & Nielsen, 2013). Others have altered standardized tasks to make the deception more convincing (e.g., Charles et al., 2013).

Interestingly, both proponents and critics of the VVG–aggression relationship have pointed the finger at demand effects. Proponents argue that they dilute the effects of manipulations, causing participants to appear less aggressive (Anderson et al., 2004, 2013). Conversely, critics suggest that any small or “tiny” effects are merely “spurious correlations” induced by demand characteristics (Ferguson & Wang, 2019, p. 1440).

## How Demand Characteristics Can Influence Outcomes in VVG Research

Research shows that gamers feel stigmatized by claims linking VVGs to aggression (Nauroth et al., 2014; Sjöström et al., 2013; Sørensen, 2016). As frequent players of VVGs are less likely to believe in a link between gaming and increased aggression than non-players (Greitemeyer, 2014), they may oppose hypotheses suggesting this relationship, especially if they believe that this is the aim of the study. This reflects coping strategies against stigmatization, such as negating stereotypes (Taylor et al., 2021). Supporting this, Bender and colleagues (2013) found that when the purpose of an aggressive cognition measure was made explicit, habitual VVG players showed reduced aggressiveness.

One plausible interpretation of these studies is that research suggesting that VVGs increase aggression is perceived as a threat to habitual players. In addition to this, if they are perceived as being aggressive, gamers may become less desirable and more likely to be excluded or discriminated against in various social contexts, thereby limiting several potential forms of freedom. Perceiving a threat to their freedoms can motivate habitual players to act in a way that would protect or restore their freedom, which would correspond to some form of reactance (Brehm, 1966; see also Rosenberg & Siegel, 2018, for an updated review of psychological reactance theory).

Hence, we reasoned that when habitual VVG players perceive the goal of the researchers to show that VVGs are positively associated with aggression, they adjust their responses to appear less aggressive. Conversely, when habitual VVG players believe that the study aims to disprove the VVG–aggression relationship, they do not tailor their responses, as researchers’ goals would not pose a threat to their identity in this case. In contrast, non-habitual VVG players should not be affected by the perception of whether the goal of the study is to show that VVGs increase aggression (or not). The potential implication would be that the relationship between VVGs and aggression may be underestimated in studies that involve habitual players and if participants are suspicious of the researchers’ goal.

## The Present Research

**Figure 1:**
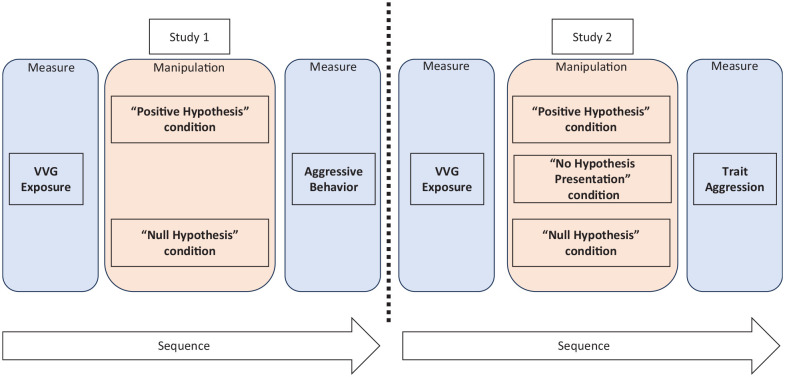
Simplified Representation of the Design of the Two Studies *Note*. This representation only depicts variables relevant to the main hypothesis. Additional variables are included in the study but not depicted in this representation.

We conducted two high-powered experiments to assess how behavioral and trait aggression measures are influenced by cues that inform on the researchers’ hypothesis. Participants were randomly assigned to conditions that manipulated how our goal was presented: either “confirming a positive relationship between VVGs and aggression” or “confirming a lack of relationship between VVGs and aggression”. A third condition in Study 2 provided no information about the research hypothesis. Both studies assessed long-term VVG exposure^2^. Study 1 measured its relationship to aggressive behavior, while Study 2 evaluated trait aggression (see Figure 1 for an illustration of our designs).

We hypothesized an interaction between the manipulated “Hypothesis Presentation” factor and VVG exposure. Specifically, we predicted that the relationship between VVG exposure and aggression would be stronger when participants believed the study aimed to disprove the VVG–aggression relationship compared to when they believed it aimed to confirm it. Pre-registrations, complete datasets, materials, and analysis codes are publicly available on Open Science Framework (OSF; https://osf.io/bv697).

## Study 1

### Method

#### Sampling Strategy

The study was conducted online. *N* = 788 participants, from 36 countries, were recruited using Prolific (www.prolific.co) from July 5, 2023, to July 11, 2023, with English as a fluent language and 95% approval ratings as filters.

The sample size was determined a priori by a power analysis conducted using R (R Core Team, 2023) on R Studio (Posit team, 2023), with the InteractionPoweR package (Baranger et al., 2023) for a two-level moderator. The power analysis was conducted assuming small effect sizes (*r* = .10) for both main effects, the interaction effect, and the correlation between the two predictor variables. A sample size of *N* = 750 was sufficient to provide 80% power (1−β err probability) to detect the interaction given these parameters (with an α err probability of .05). To account for potential exclusion of data due to any data quality issue, we increased the sample size by 5%, resulting in *N* = 787.5 (rounded to 788). Given the limited amount of existing research on this specific topic, making informed predictions about the effect sizes is challenging. The small effect sizes we assumed represent the lower bound of effect sizes that would hold practical relevance. A total of *N* = 813 participants started the study, but *n* = 25 were automatically returned to Prolific after failing attention checks (we provide evidence that failing attention checks did not generate any form of selection bias in the Supplementary Materials on the OSF repository). The final sample of *N* = 788 thus provided 81.2% power to detect the interaction.

#### Sample Description

The mean age of the sample was *M* = 28.93 (*SD* = 8.94), *n* = 350 participants were females and *n* = 438 were males^3^ (a more detailed description of the sample is provided in the Supplementary Materials on the OSF repository).

#### Experimental Manipulation

After providing baseline information about themselves (e.g., demographics and video game consumption), participants were randomly assigned to one of two conditions: the “Positive Hypothesis” condition or the “Null Hypothesis” condition. Participants were informed that the previous questions were simply to understand what type of participants were taking part in the study and that the actual experiment was beginning now. To make it credible, the design made it look like the beginning of a new study, consent to take part was asked again, and the purpose of the experiment was presented.

In the “Positive Hypothesis” condition, participants (*n* = 393) were informed that our goal was to confirm that VVG players were more aggressive than non-VVG players.

In the “Null Hypothesis” condition, participants (*n* = 395) were informed that our goal was to confirm that VVG players were not more aggressive than non-VVG players.

#### Measures^4^

##### Video Game Consumption and VVG Exposure

Participants were asked to report the average number of days per week they have spent playing games of nine video game genres, over the past 5 years. The answers ranged from 0 to 7 days. The nine genres were as follows: Action games (*M* = 2.93, *SD* = 2.25), Adventure games (*M* = 2.89, *SD* = 2.19), Action-Adventure games (*M* = 2.88, *SD* = 2.21), Role-Playing games (*M* = 2.76, *SD* = 2.21), Strategy games (*M* = 2.39, *SD* = 2.04), Simulation games (*M* = 1.87, *SD* = 1.93), Sandbox games (*M* = 1.84, *SD* = 1.94), Massively Multiplayer Online Role-Playing games (*M* = 1.81, *SD* = 2.15), and Sport games (*M* = 1.44, *SD* = 1.95).

As pre-registered, VVG Exposure (*M* = 8.70, *SD* = 5.84) was computed by calculating the sum of the frequency of game play for Action, Adventure, and Action-Adventure video games. We focused on these three genres of video games because these genres typically involve physical aggression against other players or other characters as a core and frequent aspect of the gameplay and the narrative (see Thompson & Haninger, 2001). A bifactor CFA conducted with the Lavaan package (Rosseel, 2012) using the R statistical language (R Core Team, 2023) in R Studio (Posit team, 2023) confirmed the distinctiveness of VVG exposure from a general overarching construct corresponding to the frequency of playing video games in general (the details are provided in the Supplementary Materials on the OSF repository).^5^ Genre-based measures of exposure to VVGs have been associated with aggression in the past (Busching et al., 2013; Möller & Krahé, 2009).

##### Aggressive Behavior

Aggressive behavior was measured using a modified version of the Taylor Aggression Paradigm (Taylor, 1967). Participants were informed that they were connected to another player, who was ostensibly also a participant in the study (a loading screen indicated that the participant had to wait for someone else to connect, and after a few seconds, the game ostensibly connected to another player).

In this modified version, the task consisted of seven trials, and before each trial participants had to indicate the length of a loud, uncomfortable noise blast that the other player ostensibly had to listen to if they lost (i.e., if the participant won a trial), and afterwards, they had to solve a riddle.

They were instructed to find the correct answer to each riddle and respond as quickly as possible. Participants were informed that if one player had the correct answer and the other player had the wrong answer, the one with the correct answer would win, regardless of the time taken to answer. If both players had the wrong answer, the one who responded more quickly would win. And if both players had the correct answer, then, again, the faster responder would win. This approach was used to enhance engagement and increase the credibility of losing trials, even when participants were confident in their response.

Upon winning a trial, the game indicated that the other player was being blasted with the noise. When participants lost, they were subjected to the noise blast. Trials were designed to be won or lost in a specific sequence, regardless of the answer chosen or the time taken by the participant. Participants won trials 1, 4, 5, and 7, and lost trials 2, 3, and 6. Trials were separated in three types: “Provocation” trials, Critical trials, and Filler trials. “Provocation” trials were those where the participant lost right before a winning trial: 3 and 6. Critical trials were those where the participant won immediately after losing (i.e., after provocation): 4 and 7. All other trials were filler trials: 1, 2, and 5. Trials 1 and 2 were there to allow the participants to familiarize themselves with the game, and the Trial 5 was there to increase the credibility of the game.

For each trial where the participant lost, the noise blast length was randomly selected from durations of 1 to 10 seconds. The screen displaying the message that “the other player is deciding what length the noise blast should be” had a randomly selected duration of 5, 7, or 9 seconds, to increase the credibility that the decision time for the other player was not systematically the same. As pre-registered, the cumulative value for the two critical trials serves as the aggression score. Aggression scores ranged from min = 2 to max = 20. The overall average aggression score was 8.70 (*SD* = 5.71).

##### Belief That the Researchers Wanted to Find a Positive Relationship

At the conclusion of the study, and before the debriefing, participants were asked six questions to assess their understanding of the study’s false hypotheses. They were informed that this section was designed to ensure they had correctly understood the study and that it ostensibly served as a data quality check. The values (*M* = 32.11, *SD* = 21.53) ranged from 6 (indicating a strong belief that the researchers wanted to find a null effect) to 60 (indicating a strong belief that the researchers wanted to find a positive effect).

### Results

All analyses were conducted using R (R Core Team, 2023) in R Studio (Posit team, 2023).

#### Manipulation Check

The score for the belief that the researchers wanted to find a positive relation was *M* = 13.91 (*SD* = 12.09) in the “Null Hypothesis” condition, whereas it was *M* = 50.40 (*SD* = 10.69) in the “Positive Hypothesis” condition. A Welch Two-Sample One-Sided *t*-test indicated that the effect was statistically significant and large, *t*(775.23) = −44.89, *p* < .001, Cohen’s *d* = −3.22, 95% confidence interval (CI) = [−Infinity, −3.04].

#### Confirmatory Analyses

##### The VVG Exposure–Aggression Relationship

VVG exposure was positively associated with aggressive behavior, *t*(786) = 2.52, *p* = .012, Std. β = .09, 95% CI = [.02, .16], *r* = .09.

##### The VVG Exposure × Hypothesis Presentation Moderation

**Figure 2:**
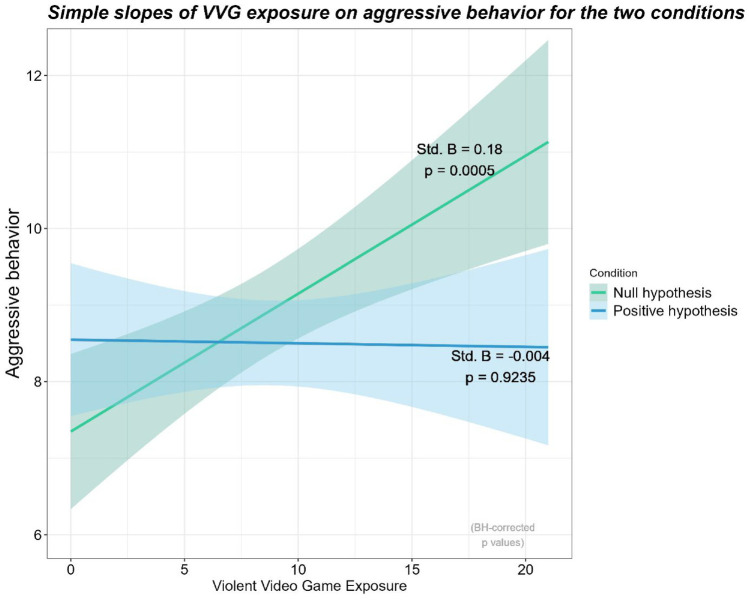
The Moderation Effect of VVG Exposure and Hypothesis Presentation on Aggressive Behavior

The moderation analysis was conducted using a linear model with ordinary least squares using the R statistical language (R Core Team, 2023) in R Studio (Posit team, 2023). The moderation was decomposed using the emmeans package (Lenth, 2023) and the Johnson–Neyman interval was computed with the interactions package (Long, 2019). As hypothesized, the moderation effect of VVG Exposure × Condition (with the “Null Hypothesis” condition as the reference level) was statistically significant, *t*(784) = −2.68, *p* = .008, Std. β = −.19, 95% CI = [−.33, −.05]. In the “Positive Hypothesis” condition, VVG exposure was not significantly associated with aggressive behavior, *t*(784) = −.096, Benjamini–Hochberg adjusted *p* = .924, Std. β = −.004, 95% CI = [−.10, .09], *r* = −.01, whereas in the “Null Hypothesis” condition, there was a significant positive relationship, *t*(784) = 3.69, Benjamini–Hochberg adjusted *p* < .001, Std. β = .18, 95% CI = [.09, .14], *r* = .18 (Figure 2).

A Johnson–Neyman Interval analysis (Bauer & Curran, 2005) indicated that only when VVG exposure was outside the [−3.31, 11.13] interval, the effect of Hypothesis Presentation on aggression was significant. The range of observed values of VVG exposure was [0.00, 21.00] and negative values were impossible, therefore indicating that only habitual VVG players (i.e., participants with VVG exposure values higher than 11.13) displayed different levels of aggression for each Hypothesis Presentation condition.

### Discussion

Our findings provide evidence for a causal effect of the way the hypothesis was presented on the behavioral aggression responses of habitual players. Unlike participants who had little or no exposure to VVGs, those at high levels of exposure showed different levels of aggressive behavior across the two conditions: they responded less aggressively in the “Positive Effect” condition than in the “Null Effect” condition. This led the relationship between VVG exposure and aggressive behavior to be nonsignificant in the “Positive Effect” condition, whereas it was significant in the “Null Effect” condition.

## Study 2

In this second experiment, we measured trait aggression rather than behavioral aggression. Furthermore, we included a third condition that had no information about the hypothesis to resemble typical VVG studies. Because no belief was artificially induced in this condition, we expected the magnitude of the relationship between VVG exposure and trait aggression to fall somewhere between the magnitude in the “Positive Effect” condition and the “Null Effect” condition.

### Method

#### Sampling Strategy

*N* = 1,182 participants from 34 countries were recruited using Prolific (www.prolific.co), with English as a fluent language and 95% approval ratings as filters, from July 14, 2023, to July 21, 2023.

The sample size was determined a priori by a power analysis conducted using R (R Core Team, 2023) on R Studio (Posit team, 2023) with the InteractionPoweR package (Baranger et al., 2023) for a three-level moderator. The power analysis was conducted assuming small effect sizes (*r* = .10) for both main effects, the interaction effect, the correlation between the two predictor variables, and assuming a reliability of .70 for our outcome measure. A sample size of *N* = 1,182 was sufficient to provide 81.88% power (1−β err probability) to detect the interaction given these parameters (with an α err probability of .05). A total of *N* = 1,220 participants started the study, but *n* = 38 failed the attention checks and were automatically returned to Prolific (we provide evidence in the Supplementary Materials on the OSF repository that failing attention checks was not related to any form of selection bias).

#### Sample Description

The mean age of the sample was *M* = 32.26 (*SD* = 10.97), *n* = 657 participants were females, *n* = 521 were males, *n* = 2 were intersex, *n* = 1 was non-binary, and *n* = 1 preferred not to say^6^ (a more detailed description of the sample is provided in the Supplementary Materials on the OSF repository).

#### Experimental Manipulation

The presentation and timing of the manipulation was identical to the first study, the only difference was that we included a third condition “No Hypothesis Presentation” (*n* = 383). In this condition, no information was provided about the researchers’ goals and hypotheses, thus mimicking how typical VVG studies are presented. The other two “Positive Hypothesis” (*n* = 401) and “Null Hypothesis” (*n* = 398) conditions were identical to the first study.

#### Measures^7^

##### Trait Aggression

All measures were identical to Study 1 with the exception of the outcome. We used the BPAQ (Buss-Perry Aggression Questionnaire, Buss & Perry, 1992) to measure trait aggression. The BPAQ is designed to assess four dimensions of trait aggression: Physical Aggression, Verbal Aggression, Anger, and Hostility, with a total of 29 items. The sequence of these items was randomized for the study. Responses were collected using a 21-level slider, representing incremental percentages of agreement from 0% to 100%, with intervals of 5%. The structure of the BPAQ in our data was confirmed with a hierarchical CFA using the Lavaan package in R (more details are provided in the Supplementary Materials in the OSF repository). Trait Aggression scores ranged from 1 to 21, with an average score of *M* = 7.15 (*SD* = 3.14).

##### VVG Exposure

We used the same measure as in Study 1. The distinctiveness of VVG exposure from a general gameplay frequency overarching construct was confirmed with a bifactor CFA conducted on Lavaan in R (more details are provided in the Supplementary Materials in the OSF repository). The average VVG exposure score was *M* = 7.45 (*SD* = 5.81).

##### Belief That the Researchers Wanted to Find a Positive Relationship

We used the same measure as in Study 1. The average belief was *M* = 33.49 (*SD* = 18.67).

### Results

#### Manipulation Check

The score for the belief that the researchers wanted to find a positive relation was *M* = 15.11 (*SD* = 13.15) in the “Null Hypothesis” condition, whereas it was *M* = 50.44 (*SD* = 10.76) in the “Positive Hypothesis” condition. In the “No Hypothesis Presentation” condition, the score was *M* = 34.84 (*SD* = 11.02).

A Welch Two-Sample One-Sided *t*-test indicated that the difference between the “Null Hypothesis” and “Positive Hypothesis” conditions was significant, *t*(764.79) = 41.55, *p* < .001, Cohen’s *d* = 3.01, 95% CI = [2.83, Infinity], indicating that the manipulation worked.

We had no a priori prediction about the third condition “No Hypothesis Presentation,” but exploratory analyses indicated that the difference with the “Positive Hypothesis” condition was significant, *t*(778.19) = 20.05, *p* < .001, Cohen’s *d* = 1.44, 95% CI = [1.30, Infinity], and so was the difference with the “Null Hypothesis” condition, *t*(764.69) = −22.76, *p* < .001, Cohen’s *d* = −1.65, 95% CI = [−Infinity, −1.51].

#### Confirmatory Analyses

##### The VVG Exposure–Trait Aggression Relationship

VVG exposure was positively associated with trait aggression, *t*(1180) = 5.13, *p* < .001, Std. β = .15, 95% CI = [.09, .20], *r* = .15.

##### The VVG Exposure × Hypothesis Presentation Moderation

The moderation effect was statistically significant and negative with the “Null Hypothesis” condition as the reference level and the “No Hypothesis” condition as the comparison level, *t*(1176) = −2.64, *p* = .008, Std. β = −.19, 95% CI = [−.33, −.05]. The moderation was also statistically significant and negative with the “Null Hypothesis” condition as the reference level and the “Positive Hypothesis” condition as the comparison level, *t*(1176) = −3.42, *p* < .001; Std. β = −.24, 95% CI = [−.38, −.10].

**Figure 3:**
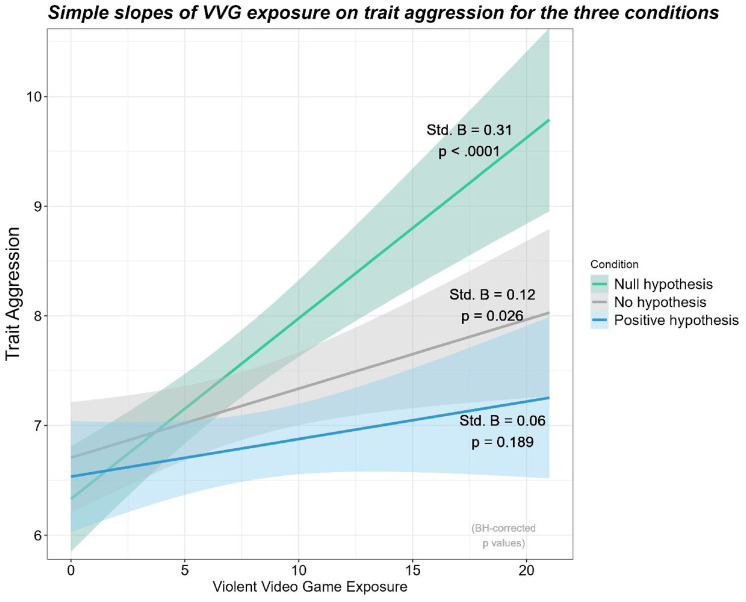
Graphical Representation of the Moderation of VVG Exposure and Hypothesis Presentation on Trait Aggression

In the “Positive Hypothesis” condition, VVG exposure was not significantly associated with trait aggression, *t*(1176) = 1.32, Benjamini–Hochberg adjusted *p* = .189, Std. β = .06, 95% CI = [−.03, .16], *r* = .07, whereas VVG exposure was positively associated with trait aggression in the “Null Hypothesis” condition, *t*(1176) = 5.89, Benjamini–Hochberg adjusted *p* < .001, Std. β = .30, 95% CI = [.20, .41], *r* = .28. In the “No Hypothesis” condition, the relationship was also positive, *t*(1176) = 2.37, Benjamini–Hochberg adjusted *p* = .027, Std. β = .12, 95% CI = [.02, .21], *r* = .12 (Figure 3).

A Johnson–Neyman Interval analysis indicated that when VVG exposure was outside the [−7.8, 5.16] interval, the effect of hypothesis presentation on trait aggression was significant. The range of observed values of VVG exposure was [0, 21], and negative values were impossible, therefore indicating that only participants with VVG exposure values higher than 5.16 would display different levels of trait aggression for each hypothesis presentation condition.

#### Exploring the “No Hypothesis Presentation” Condition

Within this condition, the belief that the researchers wanted to find a positive relation can serve as a measure of the presence of demand effects (i.e., the moderating effect of participants’ belief about the hypothesis). The belief ranged from 6 to 60, and the average score for the belief was *M* = 34.84, making it higher than the 50% threshold value of 33, *t*(382) = 3.26, *p* = .001, Cohen’s *d* = .17, 95% CI = [.07, .27]. These values suggest that without any information about the study’s goal, the average belief was that we had a positive hypothesis (i.e., with a value higher than 33).

A linear regression model with an interaction term between this belief and VVG exposure indicated a significant moderation on trait aggression, *t*(379) = −3.67, *p* < .001; Std. β = −.18, 95% CI = [−.27, −.08].

**Figure 4:**
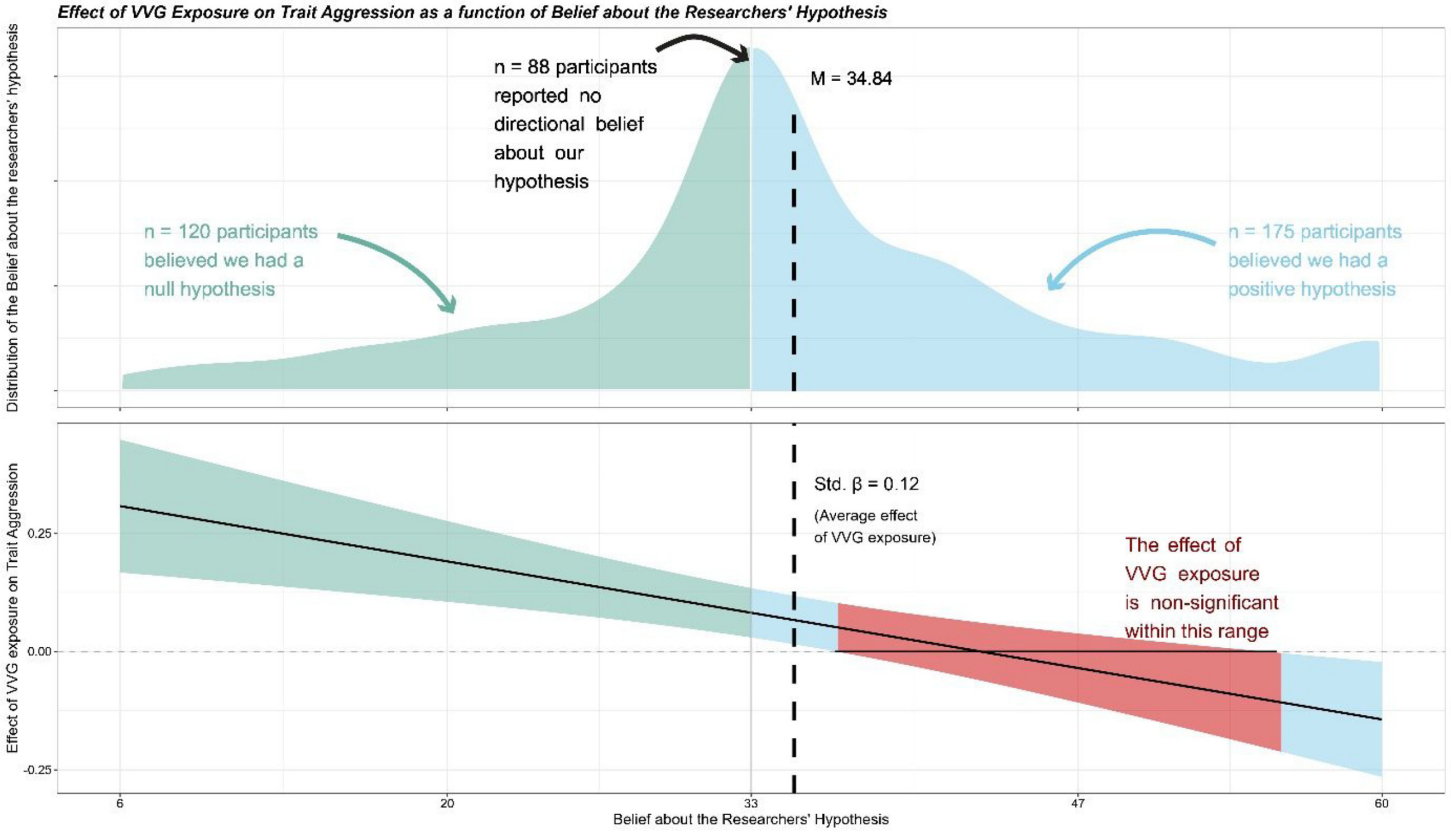
Graphical Representation of the Conditional Effect of the Belief About the Researchers’ Hypothesis on the Relationship Between VVG Exposure and Trait Aggression *Note*. The dashed vertical line represents the average belief of participants.

A Johnson–Neyman Interval analysis indicated that when the value of the belief about the researchers’ hypothesis was outside the [36.62, 55.03] interval, the effect of VVG exposure was significant (Figure 4). When the belief was below 36.62, VVG exposure positively predicted trait aggression. When the belief was higher than 55.03, VVG exposure negatively predicted trait aggression.

### Discussion

Habitual players not only adjust aggressive behaviors (Study 1) but also trait aggression responses, suggesting that even trait measures are not impervious to demand effects. Further advancing Study 1, the relationship between VVG exposure and trait aggression in the control condition was moderated by the participants’ belief about the researcher’s hypothesis. These findings suggest once again that habitual VVG players aim to appear less aggressive when they believe the study they are participating in wants to establish a positive relationship between VVGs and aggression, leading to a less pronounced relationship compared to the “Null Hypothesis” condition (as in Study 1), but also compared to the “No Hypothesis” condition (that resembles a typical VVG study).

## General Discussion

As predicted, the hypothesis presentation manipulation influenced the responses to both a behavioral aggression measure and to a trait aggression measure. In both studies, the effect emerged after a certain level of VVG exposure and amplified with increasing exposure. The demand effect we identified in our studies is, therefore, one that potentially occurs when studies include habitual players. The exploratory analyses of Study 2 provide further insight on how demand effects may occur. Even in the absence of any information about the alleged goal of the researchers, many participants are likely to believe researchers aim to link VVGs and aggression, suggesting an inherent bias in VVG research.

Together, our studies provide compelling evidence that habitual VVG players who believe that researchers want to show a positive relationship between VVGs and aggression are motivated to disprove this relationship, and that they do so by adjusting their responses to both behavioral measures of aggression and self-reported measures of trait aggression. The exploratory analysis of Study 2 indicates how participants’ beliefs can bias VVG studies toward null effects due to their “default” beliefs (i.e., that have not been influenced by information in the study presentation). Crucially, our two studies demonstrate that participant responses in VVG studies can be significantly influenced by their beliefs and information provided to them and that the tendency of habitual VVG players to alter their responses may lead to an underestimation of the VVG–aggression relationship.

These findings also contribute to the ongoing debate about the relationship between VVGs and aggression by suggesting that demand effects may play a role in the inconsistencies found across studies. Given the lack of attention given to demand effects, there may be high variability across studies in the extent to which they induce demand effects. However, the extent to which this phenomenon has actually biased the existing literature, and in what direction, remains unknown. In the hundreds of VVG studies conducted, there has been considerable variation in how VVG exposure and aggression have been operationalized, measured, or manipulated. It is therefore possible that the sensitivity of these variables to the demand effect may differ, and it should be noted that our findings do not address this issue.

More importantly, the more subtle cues that can generate beliefs about the hypothesis can vary substantially and originate from various sources on a case-by-case basis for each type of study. Apart from the different proportion of habitual players as participants, other aspects such as cover stories, how the measures are presented, attention to detail in scripting, acting or the information given, participants’ prior knowledge of the study topic or of the researchers (e.g., student may be aware of the hypotheses of researchers at their universities), and participants’ personalities are all parameters that may influence the occurrence of demand effects. For instance, the exploratory analysis of Study 2 indicates that the relationship between VVG exposure and trait aggression responses can vary considerably depending on participant belief. In our sample of Study 2 control participants, that belief had a notable degree of variability. One can imagine a multitude of potential combinations of the small cues we listed that may, in practice, subtly push participants’ beliefs in a particular direction. A more pessimistic view on the literature may lead one to consider the possibility of researchers knowingly using these subtle cues to increase their chances of getting a desired result.

The findings presented here do not diminish the significance of key methodological discussions and controversies in this field, including concerns about questionable research practices (Ballou, 2023), publication bias (Hilgard et al., 2017), and measurement (Elson et al., 2014). Instead, we argue that demand effects also contribute to the variability in observed effects. Given all the ways previously described in which demand characteristics may influence findings in VVG studies, it is important to note that our two studies make the case that in some contexts, demand effects may contribute to an underestimation of the VVG–aggression relationship. This contrasts other methodological criticism in this field which has systematically pointed at issues leading to an overestimation of the effect of VVGs. Our two experiments provide strong evidence that when participants are both suspicious and habitual VVG players, they have lower aggression levels. This suggests that the relationship between VVG exposure and aggression may be underestimated in contexts where both factors are present. However, given the potential variations in levels of suspicion and the proportion of habitual players across studies, it is not possible to conclude, based on our findings alone, that the impact of VVGs on aggression is consistently underestimated in all VVG research.

Importantly, given that habitual players are central to the field and they are the targeted demographic of many studies, to avoid potential demand effects and guarantee the validity of conclusions, researchers must turn to managing participant suspicion.^8^

It is also important to note that an alternative perspective exists regarding our approach to studying demand effects. Corneille and Lush (2023) argue that demand effects are inherently “challenging to control” due to the subtlety of cues involved (p. 84). This view implies that having explicitly manipulated the hypothesis presentation, the phenomenon we observe in our two studies should not be characterized as a demand effect. Nevertheless, our approach follows a typical method to study demand effects, as evidenced by a recent meta-analysis by Coles and Frank (2023), which examined 195 effect sizes from studies in which the hypotheses communicated to participants were manipulated.

Furthermore, research on demand effects in VVG studies should explore potential systematic differences between observational and experimental designs. Typically, between-design experiments may expose participants to less information that could influence their beliefs. However, the overall prevalence and direction of demand effects in these designs have yet to be thoroughly investigated. Nonetheless, a plausible prediction based on our results is that habitual players, if included in experimental studies, may hold beliefs and motivations that could bias the outcomes.

Finally, another important step in investigating demand effects in VVG research would be to conduct a direct empirical test of the underlying processes that lead to these patterns of responses, namely with regard to the motivation to combat the negative stigma. Further research should also directly investigate to what extent participants feel threatened as a function of their belief about the research hypothesis.

## Conclusion

In conclusion, our results reveal an ironic pattern: When the studies were presented as attempts to prove that VVGs increase aggression, the effects were not significant. However, when presented as attempts to confirm a null effect, significant relationships emerged. This puzzling and paradoxical pattern demonstrates the imperative need to be cautious, in VVG research and in psychological research overall, of subtle cues and of participant motivations that may skew results, both when designing studies and when examining findings in the published literature. Our findings serve as an example of how demand characteristics can skew the estimation of psychological effects when participants have prior knowledge of the topic being studied or a vested interest in the study results. This phenomenon threatens the validity of research on various topics in which participants become aware of expectations, including the popular rubber-hand illusion (see Lush, 2020) and sensitive topics related to politics or morality like pro-environmental behavior (see Koller et al., 2023). This growing concern regarding the influence of demand effects should motivate researchers across psychology fields to rigorously scrutinize the extent to which study outcomes are merely artifacts caused by motivated responding. This phenomenon may threaten the validity of behavioral and trait measures in many areas of psychology. Just as in VVG research, many scientific endeavors can lead us to believe we are examining a specific relationship between variables, but participants’ beliefs and motivations can introduce bias, veering us off the intended course, thereby unveiling the elaborate “game within the game” that we are truly navigating.

## Notes

1. While we highlight the role of demand characteristics that may lead to an underestimation of the relationship between VVGs and aggression, we do not claim that these influences are more substantive than other factors that may contribute to an overestimation of the effects. Thus, our work should be viewed as a complement to the broader discourse on methodological diversities and biases within VVG research.

2. It is important to note that we examined whether the correlational relationship between VVGs and aggression, rather than manipulating exposure to VVGs, would be moderated by how the goal of the study was communicated. We come back to this issue in the General Discussion.

3. We provide evidence that our main findings do not vary as a function of gender in the Supplementary Materials for Study 1 on the OSF repository.

4. In this section, we present the measures relevant to the hypothesis being tested. Several additional measures were used for exploratory purposes. All information about these measures, the corresponding data, and the results of our exploratory analyses are available on the OSF repository.

5. We provide further evidence of the performance of this measure in the Supplementary Materials on the OSF repository.

6. We provide evidence that our main findings vary as a function of gender in the Supplementary Materials for Study 2 on the OSF repository.

7. In this section, we present the measures relevant to the hypothesis being tested. Several additional measures were used for exploratory purposes. All information about these measures, the corresponding data, and the results of our exploratory analyses are available on the OSF repository.

8. We provide some guidelines in the Supplementary Materials of Study 2 for improving future VVG studies with regard to managing suspicion and improving our knowledge regarding demand effects in VVG research.
